# Four Major Psychiatric Disorders in Childhood and Early Adulthood and Siblings’ Subsequent Socioeconomic Status: A Nationwide Familial Coaggregation Cohort Study

**DOI:** 10.1192/j.eurpsy.2025.361

**Published:** 2025-08-26

**Authors:** W. Yang, K. Komulainen, R. Niemi, M. Gutvilig, P. Böckerman, M. Elovainio, C. Hakulinen

**Affiliations:** 1 University of Helsinki; 2 Labour Institute for Economic Research LABORE, Helsinki, Finland

## Abstract

**Introduction:**

Previous studies show the aggregation of major psychiatric disorders (MPDs; a combined category of schizophrenia, bipolar disorder, depression, and anxiety) among siblings. However, few studies have examined whether MPDs in childhood and early adulthood are associated with siblings’ future socioeconomic status (SES).

**Objectives:**

To assess subsequent SES outcomes among siblings of individuals with an MPD diagnosed at age 5–25.

**Methods:**

This cohort study included 54,742 full siblings, 4,490 paternal, and 4,858 maternal half siblings of individuals born in Finland between 1975–1985 with MPDs diagnosed at ages 5–25 (affected probands). We defined the reference groups as identical types of siblings of individuals without any MPD diagnosis (matched unaffected probands). The siblings of both the affected and the unaffected probands were followed from the diagnosis date of affected probands until December 31, 2020. MPD diagnoses were obtained from the Finnish Care Register. SES was measured through employment status, annual disposable income (measured in EUR), and educational achievement derived from the FOLK module of Statistic Finland. Conditional logistic regression, median regression, and Generalized Estimating Equations (GEE) models were applied to estimate the adjusted associations.

**Results:**

The median age (interquartile range, IQR) at baseline was 20 years (16–24) for full siblings, 17 years (12–26) for maternal half-siblings, and 18 years (12–26) for paternal half-siblings of the affected and unaffected probands. Compared to siblings of the unaffected probands, the odds of unemployment were 50% higher (95% CI: 1.46-1.55) in full siblings of affected probands with any MPD; this association was particularly pronounced in full siblings of an affected proband diagnosed before age 15 (aOR: 1.68, 95% CI 1.49-1.90). Full siblings of the affected probands were more likely not to attain a university degree (aOR: 1.37, 95% CI 1.33-1.41). The median annual disposable income was 1,518.3 EUR lower (95% CI: -1647.4, -1389.3) in full siblings of affected probands. Similar but weaker associations were observed in maternal and paternal half-siblings. For example, compared with the half siblings of the unaffected probands, the odds of unemployment were 29% (95%CI 1.16-1.44) and 23% (95%CI 1.10-1.38) higher in maternal and paternal half-siblings of affected probands with any MPD, respectively.

**Conclusions:**

Our findings suggest that the unfavorable socioeconomic consequences of MPDs might extend to siblings.

**Disclosure of Interest:**

None Declared

